# Association of maternal nationality with preterm birth and low birth weight rates: analysis of nationwide data in Japan from 2016 to 2020

**DOI:** 10.1186/s40748-023-00149-1

**Published:** 2023-03-08

**Authors:** Tasuku Okui, Yoko Sato, Seiichi Morokuma, Naoki Nakashima

**Affiliations:** 1 Medical Information Center, Kyushu University Hospital, 812-8582 Maidashi3-1-1 Higashi-Ku, Fukuoka City, Fukuoka Prefecture Japan; 2 Department of Health Sciences, Graduate School of Medical Sciences, Kyushu University, Fukuoka, Japan

**Keywords:** Japan, Preterm birth, Nationality, Low birth weight rate

## Abstract

**Background:**

The rate of low birth weight or preterm birth is known to vary according to the birth place of mothers. However, in Japan, studies that investigated the association between maternal nationalities and adverse birth outcomes are few. In this study, we investigated the association between maternal nationalities and adverse birth outcomes.

**Methods:**

We obtained live birth data from the Vital Statistics 2016–2020 of the Ministry of Health, Labour, and Welfare. We used data on maternal age, sex, parity, gestational age, birth weight, number of fetuses, household occupation, paternal nationality, and maternal nationality for each infant. We compared the rates of preterm birth and low birth weight at term among mothers whose nationalities were Japan, Korea, China, Philippines, Brazil, and other countries. Log binomial regression model was used to investigate the association between maternal nationality and the two birth outcomes using the other infants’ characteristics as covariates.

**Results:**

In the analysis, data on 4,290,917 singleton births were used. Mothers from Japan, Korea, China, the Philippines, Brazil, and other nations had preterm birth rates of 4.61%, 4.16%, 3.97%, 7.43%, 7.69%, and 5.61%, respectively. The low birth weight rate among Japanese mothers was 5.36% and was the highest among the maternal nationalities. Regression analysis showed that the relative risk for preterm birth among Filipino, Brazilian, and mothers from other countries (1.520, 1.329, and 1.222, respectively) was statistically significantly higher compared with Japanese mothers. In contrast, the relative risk for Korean and Chinese mothers (0.870 and 0.899, respectively) was statistically significantly lower compared with Japanese mothers. Mothers from Korea, China, the Philippines, Brazil, and other nations had a relative risk for low birth weight that was statistically significantly lower than that of Japanese mothers (0.664, 0.447, 0.867, 0.692, and 0.887, respectively).

**Conclusions:**

Support for mothers from the Philippines, Brazil, and other countries are necessary to prevent preterm birth. A future study is necessary to investigate the differences in characteristics among mothers of different nationalities in order to uncover the reason for the high risk for low birth weight among Japanese mothers.

**Supplementary Information:**

The online version contains supplementary material available at 10.1186/s40748-023-00149-1.

## Background

The rates of low birth weight or preterm birth are representative indicators of adverse birth outcomes and had been positively related with neonatal or infant mortality [1, 2]. These rates largely vary among countries [3, 4] and among the birth place of mothers even within the same country [5, 6]. In Japan, the rate of low birth weight or preterm birth is known to vary according to the maternal social characteristics [7–9]. However, studies on the association between maternal nationalities and adverse birth outcomes are few in Japan.

Over the recent decades, the number of non-Japanese people in Japan had been increasing [10], along with the increasing number of births from non-Japanese women [11]. Although the total fertility rate is not high among non-Japanese women, the reported number of births from non-Japanese women in Japan was approximately 20,000 each year [11]. A study has investigated the differences in infant and fetal mortality rates according to the nationality of mothers living in Japan, and the rates among Filipino mothers were high [12]. However, the association of maternal nationalities with the rate of low birth weight or preterm birth has not been investigated. In other countries, many studies on foreign-born or immigrant mothers and adverse birth outcomes found that native-born mothers tended to have lower risk of adverse birth outcomes [5, 6, 13, 14], but the results varied depending on the country or ethnicity [15, 16]. Non-Japanese mothers are known to have some difficulties during the perinatal period in Japan [17]. Knowing the differences in adverse birth outcomes depending on maternal nationality in Japan would enable implementation of preventive measures among the high risk population.

In this study, we investigated the association between maternal nationalities and adverse birth outcomes using the Vital Statistics in Japan.

## Methods

We used live birth data from the Vital Statistics from 2016 to 2020. The individual-level data were obtained from the Ministry of Health, Labour, and Welfare in Japan. We used data on maternal age, infant’s sex, parity, gestational age, birth weight, number of fetuses, household occupation, paternal nationality, and maternal nationality for each infant. Household occupations were classified as farmer, self-employed, full-time worker 1, full-time worker 2, other occupations, and unemployed. Full-time worker 1 meant workers of a company with < 100 employees, and full-time worker 2 meant public servants and workers or board members of a company with ≥ 100 employees. Japan, Korea, China, Philippines, Brazil, Thailand, the United States, the United Kingdom, Peru, and other countries were available as nationalities of fathers and others in the data. Any combinations of maternal and paternal nationalities between couples were included in the analysis, and non-Japanese couples were also included in the analysis.

We used only singleton births, and illegitimate infants were not included, because paternal nationalities were included in the analysis. Maternal age was grouped into < 20 years, 20–24 years, 25–29 years, 30–34 years (reference), 35–39 years, and ≥ 40 years [18]. Parity was classified into primiparous and multiparous. Births with maternal nationalities from Thailand, the United States, the United Kingdom, and Peru were small in number and were included into the group of other countries in the analysis. Preterm birth was defined as gestational age of ≤ 36 weeks. Low birth weight was defined as < 2,500 g. In this study, the rate of low birth weight at term was calculated according to previous studies [19, 20].

For each maternal nationality, we counted the number of births and the rates of preterm birth and low birth weight by the infants’ characteristics. Log binomial regression model was used to investigate the association between the two birth outcomes and maternal nationality using the other infants’ characteristics (maternal age, infant’s sex, parity, household occupation, paternal nationality) as covariates. The traits of those other infants were included since they may have an impact on results and their distribution may vary based on the nationality of the mother. Log binomial regression model is a regression model used for binary outcome [21]. Relative risk (RR), its 95% confidence interval (CI), and p value were calculated for each maternal nationality; a p value of < 0.05 was judged as statistically significant.

In this study, complete-case analysis was carried out. An imputation utilizing the hot-deck imputation approach was also utilized as a sensitivity analysis [22]. With this technique, a missing value from an observation was substituted with another observation’s value whose values for non-missing variables were comparable to those of the missing value observation. All statistical analyses were conducted using R4.1.3 (https://www.r-project.org/). The statistics shown in this study were processed by the authors using the Vital Statistics data obtained from the Ministry of Health, Labour, and Welfare, and these are different from the statistics published by the Ministry.

## Results

**Fig. 1:**
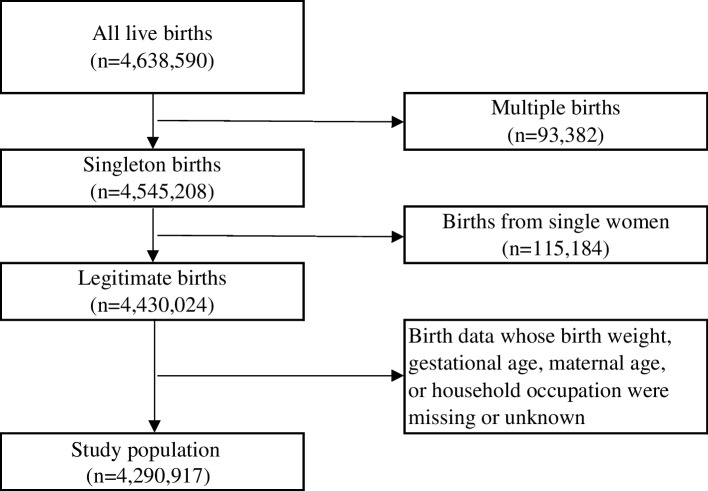
Flowchart of selection of the study population

Figure 1 shows the flowchart of selection of the study population. After removing cases with missing data, the data of 4,290,917 births were used in the analysis.

Table 1 shows the number of births by the infants’ characteristics and maternal nationality. The largest number of births was from Japanese mothers (4,182,823 births), followed by Chinese mothers (41,041 births).

**Table 1:** Number of births by the infants’ characteristics and maternal nationality

	Maternal Nationality					
	Japan	Korea	China	Philippines	Brazil	Other countries
Total	4,182,823	9,669	41,041	12,391	6,956	38,037
Maternal age group						
19 years or less	28,648	23	49	88	96	241
20–24 years	330,369	347	1,798	1,451	938	5,022
25–29 years	1,079,748	1,668	11,657	3,280	1,853	13,733
30–34 years	1,538,384	3,732	17,499	3,828	2,100	12,026
35–39 years	967,597	3,045	8,566	2,939	1,542	5,807
40 years or more	238,077	854	1,472	805	427	1,208
Sex						
Male	2,145,282	4,934	21,248	6,329	3,552	19,548
Female	2,037,541	4,735	19,793	6,062	3,404	18,489
Parity						
Primiparous	1,949,391	4,610	21,661	3,749	2,371	19,506
Multiparous	2,233,432	5,059	19,380	8,642	4,585	18,531
Household occupation						
Farmer	48,699	47	211	164	21	255
Self-employed	297,958	1,542	5,148	1,404	344	3,500
Full-time worker 1	1,377,877	3,713	16,372	4,600	1,937	13,884
Full-time worker 2	2,067,384	3,486	14,855	4,221	3,640	13,722
Other occupations	357,820	729	3,064	1,308	808	3,957
Unemployed	33,085	152	1,391	694	206	2,719
Paternal nationality						
Japan	4,139,145	6,456	14,046	7,555	1,306	10,428
Korea	9,945	2,907	161	25	7	149
China	5,879	66	26,318	25	7	166
Philippines	1,408	8	12	4,111	16	44
Brazil	2,079	4	31	245	5,406	364
Other countries	24,367	228	473	430	214	26,886
Birth weight						
1499 g or less	23,731	52	230	142	69	320
1500–2499 g	312,682	507	1,387	885	420	2,206
2500 g or more	3,846,410	9,110	39,424	11,364	6,467	35,511
Gestational age						
36 weeks or less	192,766	402	1,628	921	535	2,134
37 weeks or more	3,990,057	9,267	39,413	11,470	6,421	35,903

Table 2 shows the preterm birth rate by the infants’ characteristics and maternal nationality. The preterm birth rate for Chinese women (3.97%) was the smallest among the maternal nationalities. The rates for Filipino and Brazilian mothers were particularly high (7.43% and 7.69%, respectively), compared with those for mothers from the other countries. In addition, the preterm birth rate tended to be high in older mothers and was larger in male infants than in female infants, regardless of maternal nationality.

**Table 2:** Preterm birth rate (%) by the infants’ characteristics and maternal nationality

	Maternal Nationality					
	Japan	Korea	China	Philippines	Brazil	Other countries
Total	4.61	4.16	3.97	7.43	7.69	5.61
Maternal age group						
19 years or less	5.18	8.70	0.00	9.09	5.21	7.88
20–24 years	4.06	2.88	2.45	4.55	4.90	4.94
25–29 years	3.93	3.42	3.33	6.16	6.31	4.89
30–34 years	4.40	3.75	3.82	7.63	8.05	5.79
35–39 years	5.35	4.50	5.11	9.19	9.79	6.63
40 years or more	6.77	6.56	6.05	10.31	11.01	9.52
Sex						
Male	5.19	4.48	4.36	8.28	8.56	6.24
Female	3.99	3.82	3.54	6.55	6.79	4.95
Parity						
Primiparous	4.52	3.71	3.78	7.47	7.38	5.24
Multiparous	4.69	4.57	4.17	7.42	7.85	6.00
Household occupation						
Farmer	4.75	4.26	8.06	6.10	0.00	4.71
Self-employed	4.80	4.99	3.69	7.48	8.14	6.26
Full-time worker 1	4.72	4.34	4.06	7.57	7.80	5.56
Full-time worker 2	4.48	3.76	4.03	7.15	7.47	5.41
Other occupations	4.66	3.02	3.46	7.72	8.17	6.07
Unemployed	5.79	5.92	3.81	7.93	8.74	5.48
Paternal nationality						
Japan	4.61	4.38	4.21	7.16	5.36	5.20
Korea	4.62	3.61	4.35	4.00	14.29	6.04
China	4.35	6.06	3.84	4.00	0.00	3.01
Philippines	4.83	0.00	0.00	7.78	6.25	9.09
Brazil	6.06	0.00	6.45	8.16	8.21	7.69
Other countries	4.92	4.39	3.59	8.84	8.88	5.75

Table 3 shows the rate of low birth weight at term by the infants’ characteristics and maternal nationality. The low birth weight rate was the highest in Japanese mothers (5.36%) and was the lowest in Chinese mothers (1.90%).

**Table 3:** Low birth weight rate at term (%) by infant’s characteristics and maternal nationality

	Maternal Nationality					
	Japan	Korea	China	Philippines	Brazil	Other countries
Total	5.36	3.39	1.90	4.41	3.22	3.68
Maternal age group						
19 years or less	5.74	9.52	0.00	6.25	1.10	5.86
20–24 years	5.37	2.67	1.43	3.61	2.91	4.44
25–29 years	5.19	3.66	1.68	4.78	3.46	3.73
30–34 years	5.20	3.20	1.85	4.38	3.26	3.68
35–39 years	5.54	3.27	2.28	4.16	3.45	2.90
40 years or more	6.36	4.26	2.82	5.26	2.37	3.11
Sex						
Male	4.15	2.55	1.50	3.53	2.56	3.02
Female	6.61	4.26	2.34	5.31	3.91	4.36
Parity						
Primiparous	6.01	3.58	2.11	6.34	4.10	4.50
Multiparous	4.79	3.21	1.67	3.57	2.77	2.81
Household occupation						
Farmer	5.04	8.89	1.03	2.60	0.00	1.65
Self-employed	5.26	4.03	1.61	4.46	2.22	4.08
Full-time worker 1	5.52	3.46	1.85	4.44	3.42	3.92
Full-time worker 2	5.27	3.04	2.02	4.08	3.30	3.54
Other occupations	5.26	3.11	2.27	4.97	3.10	3.47
Unemployed	6.47	2.80	1.72	5.48	2.66	3.11
Paternal nationality						
Japan	5.37	3.89	2.58	4.63	3.64	3.36
Korea	4.45	2.46	0.00	8.33	0.00	4.29
China	3.82	0.00	1.56	8.33	0.00	5.59
Philippines	5.52	12.50	0.00	4.12	0.00	10.00
Brazil	4.71	0.00	6.90	5.33	3.18	2.38
Other countries	3.19	1.83	1.32	2.30	2.05	3.79

Table 4 shows the results of the regression analysis on the association of maternal nationality with preterm birth and low birth weight at term. Regression analysis showed that the RR for preterm birth among Filipino, Brazilian, and mothers from other countries (1.520, 1.329, and 1.222, respectively) was statistically significantly higher compared with Japanese mothers. In contrast, the relative risk for Korean and Chinese mothers (0.870 and 0.899, respectively) was statistically significantly lower compared with Japanese mothers. For low birth weight, the RR for Korean, Chinese, Filipino, Brazilian, and other countries’ mothers (0.664, 0.447, 0.867, 0.692, and 0.887, respectively) was statistically significantly lower compared with Japanese mothers.

**Table 4:** Regression analysis on the association of maternal nationality with preterm birth and low birth weight at term

	Preterm birth		Low birth weight at term	
Maternal nationality	RR (95% CI)*	*p*-value	RR (95% CI)*	*p*-value
Japan	Reference		Reference	
Korea	0.870 (0.788, 0.960)	0.006	0.664 (0.594, 0.742)	< 0.001
China	0.899 (0.841, 0.960)	0.002	0.447 (0.410, 0.488)	< 0.001
Philippines	1.520 (1.413, 1.637)	< 0.001	0.867 (0.788, 0.956)	0.004
Brazil	1.329 (1.170, 1.511)	< 0.001	0.692 (0.580, 0.826)	< 0.001
Other countries	1.222 (1.159, 1.287)	< 0.001	0.887 (0.833, 0.945)	< 0.001

*RR* Relative risk, *CI* Confidence interval

^*^Maternal age, sex, parity, household occupation, and paternal nationality were adjusted

The results of the regression analysis utilizing an imputation method on the relationship between mother nationality and preterm delivery and low birth weight at term are shown in the supplemental table. The results were consistent with the primary analysis.

## Discussion

This study using the Vital Statistics in Japan revealed an association between maternal nationality and adverse birth outcomes. Compared with Japanese women, Filipino, Brazilian, and mothers from other countries had higher risk of preterm birth. On the other hand, the risk for low birth weight at term was higher in Japanese women than in mothers from the other countries. There were possible reasons and implications of the results.

In a previous study, Filipinos in Japan were reported to have the highest infant and fetal mortality rates [12]. A similar high risk among mothers from the Philippines has been observed in Canada and Korea [5, 23]. In addition, in this study, a high risk for preterm birth was observed among mothers from Brazil and other countries. The particularly high proportion of caesarean sections affects the preterm birth rate in Brazil [24]. Additionally, the preterm birth rate was reported to be lower in Japan than the worldwide prevalence [25] and was higher in Brazil and Philippines than in Japan [26, 27]. Therefore, the difference in preterm birth rates among countries is possibly related with the difference depending on maternal nationalities in Japan. In addition, foreign mothers had been known to have difficulty in understanding health services in Japan or in communicating with others using Japanese language [17, 28]. Moreover, according to an ecological study in Japan, the percentage of non-Japanese nationalities was positively associated with the delay or lack of utilization prenatal care [29], which is known to be effective for reduction of preterm birth [30]. These factors may explain the high risk in non-Japanese mothers.

In contrast, Korean and Chinese mothers had lower risk, compared with Japanese women. Although the reason for the lower risk is uncertain, the reported preterm birth rates in Korea and China were not higher, compared with that in Japan [5, 31]. In addition, China and Korea are neighboring countries of Japan, and the population of Chinese and Koreans in Japan are higher, compared with that of Filipinos or Brazilians [10]. Therefore, people from these countries might have less psychological stress compared with those from the other countries. Another possible reason is the lower smoking prevalence among women in China and Korea than in Japan [32–34]; and a similar difference might exist among mothers in Japan.

Compared with mothers from other nationalities, Japanese women had higher risk of delivering infants of low birth weight. This result was contrary to previous reports that foreign-born or immigrant women tended have adverse birth outcomes in other countries [5, 6, 35]. The estimated worldwide prevalence of low birth weight is higher, compared with the rate in Japan [3], and non-Japanese mothers have some difficulties in childbirth in Japan. However, the relative risk for low birth weight at term was lower in non-Japanese mothers than in Japanese women. Japanese mothers’ physical traits, such as their low body mass index (BMI) and short stature, is one of the assumed causes, as evidenced by studies conducted in other nations [36–38]. Actually, a similar trend of Japanese mothers having higher risk, compared with white mothers or mothers from Korea, China, and the Philippines, was observed in the United States [36, 37]. In addition, the birth weight of infants was reported to be lower in Japanese than in other ethnicities [38]. Possible reasons for the relatively high SGA risk in Japanese mothers had been maternal height, pre-pregnancy weight, and gestational weight gain [36]. In Japan, a low birth weight rate had been known to have an increasing trend from the late twentieth century to the year 2010 [39, 40], and a decrease in birth weight was also observed [41]. Increase in the number of underweight Japanese women has been pointed out as a reason for these birth outcomes [40]. In Japan, a low pre-pregnancy BMI was found to be a major risk factor for low birth weight rate [42, 43]. According to the National Health and Nutrition Survey in 2019, the percentage of underweight (BMI < 18.5) women was 20.7% among the 20–29 years age group and 16.4% among the 30–39 years age group [44]; these values were higher compared with those reported in Korea and China [45–47]. Therefore, the high prevalence of underweight young women in Japan might be one of the reasons for the results of this study.

This study found that women whose nationality was from the Philippines, Brazil, and other countries had relatively high risk of preterm birth rate in Japan. As an implication, unawareness of the available health services in Japan could make consults for prenatal care difficult among non-Japanese women. In that case, more community intervention by public health nurses to connect medical institutions with local non-Japanese residents might be needed. In addition, an epidemiological study on the differences in health behaviors and statuses among maternal nationalities is necessary to uncover the reason for the high risk of low birth weight among Japanese women. Additionally, governments should be aware of the findings about low birth weight since they can make it known to the general population. This could help Japanese women who are expecting to ameliorate their lifestyles. Local governments can also take measures in order for non-Japanese mothers to participate prenatal care or consult a physician. Obstetricians are also advised to be aware of the results because they can speak with both Japanese and non-Japanese moms and warn them of the risks.

There were some limitations in this study. Some major characteristics of pregnant women, such as household income, education level, utilization of prenatal care, and BMI, could not be obtained, because the Vital Statistics data were used in this study. Investigating these factors in a future epidemiological study may help specify the reason for disparities. In addition, period of stay in Japan or Japanese language skill might need to be scrutinized.

## Conclusions

This study based on the Vital Statistics in Japan revealed an association between maternal nationality and adverse birth outcomes. The risk for preterm birth was significantly higher in Filipino, Brazilian, and mothers from other countries than in Japanese mothers but was significantly lower in Chinese and Korean mothers than in Japanese mothers. On the other hand, the risk for low birth weight in Japanese mothers was higher, compared with that in mothers from all the other countries.

## Supplementary Information

**Additional file 1:** **Supplementary Table.** The results of the regression analysis using an imputation method on the association of maternal nationality with preterm birth and low birth weight at term.

## Data Availability

The data used in this study were obtained from the Ministry of health, Labour, and Welfare in Japan.

## Abbreviations

BMI: Body mass index
RR: Relative risk
CI: Confidence interval
