# Supplementary material for: Association of maternal nationality with preterm birth and low birth weight rates: analysis of nationwide data in Japan from 2016 to 2020
Source: Matern Health Neonatol Perinatol. 2023 Mar 8;9:3. doi: 10.1186/s40748-023-00149-1 (PMC9993667; doi:10.1186/s40748-023-00149-1)
Supplement: Supplementary file 1 — Additional file 1: Supplementary Table. The results of the regression analysis using an imputation method on the association of maternal nationality with preterm birth and low birth weight at term. [file 40748_2023_149_MOESM1_ESM.docx]

Supplementary table: The results of the regression analysis using an imputation method on the association of maternal nationality with preterm birth and low birth weight at term.

|  | Preterm birth | | Low birth weight at term | |
| --- | --- | --- | --- | --- |
| Maternal nationality | RR (95% CI)* | p-value | RR (95% CI)* | p-value |
| Japan | Reference |  | Reference |  |
| Korea | 0.883 (0.803, 0.971) | 0.01 | 0.682 (0.613, 0.758) | <0.001 |
| China | 0.891 (0.836, 0.951) | <0.001 | 0.445 (0.408, 0.485) | <0.001 |
| Philippines | 1.532 (1.427, 1.646) | <0.001 | 0.880 (0.801, 0.966) | 0.007 |
| Brazil | 1.302 (1.148, 1.476) | <0.001 | 0.671 (0.564, 0.799) | <0.001 |
| Other countries | 1.219 (1.159, 1.282) | <0.001 | 0.895 (0.842, 0.951) | <0.001 |
| RR, relative risk; CI, confidence interval | | | | |
| *Maternal age, sex, parity, household occupation, and paternal nationality were adjusted | | | |  |
